# What Is Recent in Pancreatic Cancer Immunotherapy?

**DOI:** 10.1155/2013/492372

**Published:** 2012-12-26

**Authors:** Elena Niccolai, Domenico Prisco, Mario Milco D'Elios, Amedeo Amedei

**Affiliations:** ^1^Department of Internal Medicine, University of Florence and Patologia Medica Unit Department of Biomedicine, Azienda Ospedaliero-Universitaria Careggi, 50134 Florence, Italy; ^2^Department of Medical and Surgical Critical Care, University of Florence and Patologia Medica Unit Department of Biomedicine, Azienda Ospedaliero Universitaria Careggi, 50134 Florence, Italy; ^3^Center of Oncologic Minimally Invasive Surgery, University of Florence, Largo Brambilla 3, 50134 Florence, Italy; ^4^Division of Immunology, Department of Internal Medicine, University of Florence, Viale Pieraccini, 6, 50134 Florence, Italy

## Abstract

Pancreatic cancer (PC) represents an unresolved therapeutic challenge, due to the poor prognosis and the reduced response to currently available treatments. Pancreatic cancer is the most lethal type of digestive cancers, with a median survival of 4–6 months. Only a small proportion of PC patients is curative by surgical resection, whilst standard chemotherapy for patients in advanced disease generates only modest effects with considerable toxic damages. Thus, new therapeutic approaches, specially specific treatments such as immunotherapy, are needed. In this paper we analyze recent preclinical and clinical efforts towards immunotherapy of pancreatic cancer, including passive and active immunotherapy approaches, designed to target pancreatic-cancer-associated antigens and to elicit an antitumor response *in vivo*.

## 1. Introduction

Pancreatic cancer (PC) represents an unresolved therapeutic challenge, due to the poor prognosis and the reduced response to currently available treatments. Pancreatic cancer is the most lethal type of digestive cancers, with a median survival (MS) of 4–6 months [1]. There are three principal PC types: ductal adenocarcinoma, neuroendocrine tumors (rare), and cystic neoplasm (less than 1% of pancreatic cancers) [1]. Pancreatic ductal adenocarcinoma accounts for 90% of cancers of the pancreas and has the poorest outcome, representing the 4th most common cause of cancer-related death among men and women [2].

The only potentially curative therapy for pancreatic cancer is surgical resection. Unfortunately, only 20% PC patients are resectable at the time of diagnosis, and among those patients who undergo resection and have tumor-free margins, the 5-year survival rate after surgery is 10% to 25% [3]. Gemcitabine, with or without erlotinib, represents the standard chemotherapy but the benefit is only modest, and most patients do not survive longer than 6 months [4, 5]. 

Development of novel agents and approaches is urgently needed in conjunction with improvement in access to clinical trials for patients. Since there are different evidences that pancreatic adenocarcinomas elicit antitumor immune responses [6–9] specific immunotherapy could be of great importance in the PC treatment. In support of the PC-specific immunotherapy approaches there are numerous data showing how PC patients generate B and T cells specific to antigens expressed on autologous pancreatic tumor cells [10–12], such as Wilms' tumor gene 1 (WT1) (75%) [13], mucin 1 (MUC1) (over 85%) [14], human telomerase reverse transcriptase (hTERT) (88%) [15], mutated K-RAS (73%) [16], survivin (77%) [17], carcinoembryonic antigen (CEA) (over 90%) [18], HER-2/neu (61.2%) [19], p53 (67%) [20], and *α*-enolase [21]. Furthermore, the analysis of immune infiltrates in human tumors has demonstrated a positive correlation between prognosis and presence of humoral response to pancreatic antigens (MUC-1 and mesothelin) [8, 9, 22] or of tumor-infiltrating T cells [23]. 

In this paper we analyze recent preclinical and clinical efforts towards immunotherapy of pancreatic cancer, including passive immunotherapy approaches, such as the use of antibodies or effector cells generated *in vitro*, and active immunotherapic strategies, whose goal is to stimulate an antitumor response *in vivo*, by means of vaccination.

## 2. Passive Immunotherapy

### 2.1. Humoral Immunity: The Role of Monoclonal Antibodies

Specific recognition and elimination of pathological organisms or malignant cells by antibodies were proposed over a century ago by Paul Ehrlich, who is credited for conceptualizing the “magic bullet” theory of targeted therapy. Over the past 30 years, antibody cancer therapeutics have been developed and used clinically in aneffort to realize the potential of targeted therapy. Antibodies can target antigens differentially expressed in tumor cells (tumor-associated antigens (TAAs)) or can be used to block molecules involved in cancer progression or angiogenesis. The immunoglobulins can invoke tumor cell death by blocking ligand-receptor growth and survival pathways. In addition, innate immune effector mechanisms: antibody-dependent cellular cytotoxicity (ADCC), complement-mediated cytotoxicity (CMC), and antibody-dependent cellular phagocytosis (ADCP), are emerging as equally important [24].

Although unconjugated antibodies have had efficacy, molecular genetics and chemical modifications to monoclonal antibodies (mAbs) have advanced their clinical utility. For example, modification of immune effector engagement has improved pharmacokinetic profiles, and conjugating cytotoxic agents to mAbs has enhanced targeted therapeutic delivery to tumors. The increasing facility of antibody modifications has made it possible to construct diverse and efficacious mAb-based therapeutics.

The humoral immune response to mesothelin has been found to be a favorable prognostic factor for pancreatic cancer [8, 22, 25, 26]. Mesothelin is a 40 kDa protein present in normal mesothelial cells of the pericardium, pleura, and peritoneum, but overexpressed in mesotheliomas ovarian cancers [27] and detected in 90–100% of pancreatic adenocarcinomas [28, 29]. Different antibodies to mesothelin have been studied and in particular SS1P, a murine single-chain Fv, specific for human mesothelin, which has been fused to PE38, a 38 kDa portion of *Pseudomonas *exotoxin A (PE-A). After binding to mesothelin and subsequent internalization into cells, it inhibits protein synthesis and results in apoptosis [30]. In phase I clinical studies SS1P was found to be well tolerated, with self-limiting pleuritis as the dose-limiting toxicity. Also, the administration of a version of SS1P with releasable PEGylation resulted in complete regression of a mesothelin-expressing human carcinoma in mice with only a single dose [30–32]. MORAb-009, a monoclonal antibody against mesothelin, is being tested in a phase I trial of 11 patients (three with pancreatic cancer) [33]. One of them who had previously progressed on gemcitabine showed disease stabilization on computed tomography (CT) and a drop in CA19-9 (carbohydrate antigen 19-9). Two fully human, antihuman mesothelin antibodies, M912 and HN1, have been developed, which bind mesothelin-positive cells and result in their lysis via ADCC [34, 35]. Similar to SS1P, HN1 has been fused to truncated PE-A immunotoxin, although its binding site on mesothelin probably binds a distinct but overlapping epitope to that of SS1P [35].

MUC1 (mucin-1, CD227) is a polymorphic, glycosylated type I transmembrane protein present in glandular epithelium of different tissues (pancreas, breast, lung) and overexpressed (aberrantly glycosylated) in 90% of pancreatic cancers [36, 37]. It inhibits cell-cell and cell-stroma interactions and functions as a signal transducer in the cancer progression, including tumor invasion and metastasis [38]. Evidences suggest that circulating anti-MUC1-IgG is a favorable prognostic factor for pancreatic cancer [22]. Downregulation of MUC1 expression in human PC cell line S2-013 by RNAi significantly decreased proliferation *in vitro *and in nude mice [39]. In a murine model, the use of MUC1-specific 90Yttrium-labelled moAb PAM4 in combination with gemcitabine as a radiosensitiser [40] increased inhibition of tumor growth and prolonged animal survival. To date, it is undergoing phase I trial for stage III or IV PC patients. 

*In vitro *study showed that 213Bi-C595 was specifically cytotoxic to MUC1-expressing PC cells in a concentration-dependent manner compared to controls. 213Bi-C595 is a moAb targeting the protein core of MUC1, conjugated with the *α*-particle-emitting ^213^bismuth [37]. 

PankoMab (Glycotope, Germany) is a murine anti-human MUC-1 antibody that binds to a carbohydrate-induced conformational tumor epitope of MUC-1, greatly increasing its tumor specificity [41]. PankoMab can induce ADCC of MUC-1 positive cells and can also induce death following internalization by inhibition of RNA polymerase when linked to *β*-amanitin. The humanized version of PankoMab has been shown to react to the tumor expressed MUC-1 in multiple human carcinomas, although no clinical trials have been published [42].

The epidermal growth factor receptor 2 (HER2), a transmembrane receptor tyrosine kinase, is overexpressed in up to 45% of pancreatic cancer. An anti-Her-2/neu antibody, known as Herceptin (Genentech Inc., CA, USA) or trastuzumab, has been used with some success to treat PC murine models. Treatments with trastuzumab prolonged survival and reduced liver metastasis in nude mice orthotopically challenged with human pancreatic tumor cell lines that expressed Her-2/neu at low levels. The pancreatic lines were sensitive to ADCC lysis by trastuzumab *in vitro *[43]. Similar results were found when nude mice (challenged with Her-2/neu high expressing human PC cell lines) were treated with both trastuzumab and 5-fluorouracil [44]. The combination of treatments significantly inhibited tumor growth compared with either treatment alone. When combined with matuzumab, an anti-EGFR antibody, trastuzumab treatment, resulted in inhibited PC growth in a nude mouse [45]. Also, this combined treatment was more effective than treatment with either antibody alone or combined with gemcitabine [46]. 

Carcinoembryonic antigen (CEA), a member of a family of cell surface glycoproteins involved in cell adhesion, is frequently overexpressed in various types of human cancers. Many anti-CEA antibodies have been used for immunotherapy, such as hMN-14 (labetuzumab), which has been shown to induce ADCC *in vitro *with CEA^+^ colon tumor cells and inhibited growth of lung metastases in nude mice [47]. A phase I/II trial with hMN-14 in PC patients has been completed but the results have not been published [48].

EGFR is a transmembrane glycoprotein receptor, overexpressed in 90% of pancreatic tumors [49], which induces tumor cell proliferation and neovascularization; also this expression is associated with worse prognosis [50, 51]. Blocking EGFR signaling decreases growth and metastasis of pancreatic tumor in animal models and enhances the effects of gemcitabine [52, 53]. 

Cetuximab (Erbitux or IMC-C225) is a chimeric monoclonal antibody generated from fusion of the variable region of the murine anti-EGFR monoclonal antibody M225 and the human IgG1 constant region. Promising laboratory results have led cetuximab to be tested in clinical trials. A phase III randomized study by Southwestern Oncology Group (SWOG) tested the efficacy of cetuximab and gemcitabine combination in patients with advanced PC. The median survival was 6 months in the gemcitabine arm and 6.5 months in the combination arm for an overall hazard ratio (HR) of 1.09 (*P* = 0.14). The corresponding progression free survival was 3 months and 3.5 months, respectively. The study failed to demonstrate a clinically significant advantage of the addition of cetuximab to gemcitabine [54]. In an ongoing phase II trial with trimodal therapy of cetuximab, gemcitabine and intensity modulated radiotherapy (IMRT) for patients with advanced PC; there was no increase in toxicity profile [55]. One-year survival was 57% while median survival has not been reached. 

Matuzumab (EMD72000) is a humanized IgG1 monoclonal antibody to the human EGFR. Laboratory studies have shown promising inhibitory effects on tumor growth and angiogenesis, including L3.6pl in an orthotopic rat model [56]. In a phase I study of combined treatment with matuzumab and gemcitabine, eight out of 12 patients with advanced pancreatic adenocarcinoma showed partial response or stable disease [57]. 

Vascular endothelial growth factor (VEGF) plays a pivotal role in the control of angiogenesis, tumor growth, and metastasis [58]. VEGF and its receptors are overexpressed in PC and have been demonstrated to be a poor prognostic factor. There is suggestion that elevated serum VEGF levels correlate with tumor stage, disease recurrence, and survival [59]. Development of therapeutic strategies directed towards the VEGF mediated signaling axis has been extensively tested in patients with advanced PC. 

Bevacizumab (Avastin) is a recombinant humanized anti-VEGF monoclonal antibody. A pilot study demonstrated that bevacizumab, when added to gemcitabine in patients with metastatic PC, resulted in a significant improvement in response, survival, and progression-free survival [60]. This was immediately followed by a phase III trial by CALGB comparing gemcitabine plus bevacizumab to gemcitabine plus placebo and showing no benefit for bevacizumab addition [61]. The AviTa phase III trial that examined treatment with gemcitabine plus erlotinib with either bevacizumab or placebo has been closed. Bevacizumab, however, may have a role in palliative treatment of chemotherapy-resistant PC. In a case report, a patient with stage IV disease initially unresponsive to gemcitabine, 5-FU, irinotecan, and cisplatin subsequently responded with the addition of bevacizumab [62].

### 2.2. Cellular Mediated Immunity: Adoptive T Cell Transfer

Adoptive T cell transfer is a form of immunotherapy in which patient's own T cells are expanded and reinfused into the patient. In particular, this method involves harvesting the patient's peripheral blood T lymphocytes, stimulating and expanding the autologous tumor-reactive T cells using IL-2 and CD3-specific antibody, before subsequently transferring them back into the patient. Adoptive T cell therapy depends on the ability to optimally select or genetically engineer cells with targeted antigen specificity and then to induce the cell proliferation preserving their effector function and engraftment and homing abilities. Currently, there are no FDA-approved adoptive T cell therapy protocols for cancer, but T cell therapies have shown activity in mice models and in selected clinical applications. For example, adoptive transfer of telomerase-specific T cells was studied in a syngeneic PC murine model [63]. T cells were produced *in vitro *by coculturing human lymphocytes with telomerase peptide-pulsed dendritic cells (DCs) or *in vivo *by injection of peptide with adjuvant into C57BL/6 mice. Telomerase is a reverse transcriptase that contains an RNA template used to synthesize telomeric repeats onto chromosomal ends. Activation of telomerase and its maintenance of telomeres play a role in immortalization of human cancer cells, as telomeres shrink after each cell division [64]. Telomerase activity is found in 92–95% of pancreatic cancers [65, 66] and is associated with increased potential of invasion and metastasis and poor prognosis [67, 68]. Upregulation of telomerase may also be responsible for the development of chemotherapy resistance [69]. Animals treated with these T cells showed significantly delayed disease progression [63]. 

Adoptive transfer of MUC1-specific cytotoxic T-lymphocytes (CTLs) was able to completely eradicate MUC1-expressing tumors in mice [70]. In this perspective, in a clinical study, MUC-1-specific autologous T cells, isolated from patient PBMCs (peripheral blood mononuclear cells), were expanded by incubation with an MUC-1-presenting cell line prior to administration in PC patients. The mean survival time for unresectable patients in this study was 5 months [71]. However, patients with resectable pancreatic cancer had 1-, 2-, and 3-year survival rates of 83.3, 32.4, and 19.4%, respectively, and a mean survival time of 17.8 months. In a similar study, Kondo et al. isolated adherent cells from patient PBMCs to generate mature DCs that were then pulsed with MUC-1 peptide. The pulsed DCs were administered, along with autologous expanded MUC-1-specific T cells, to patients with unresectable or recurrent pancreatic cancer. Remarkably, a complete response was observed in one patient with lung metastases, and the MS time of the whole group was 9.8 months, suggesting that the addition of pulsed DCs may have improved the outcome [72].

## 3. Active Immunotherapy: Vaccine Strategies

Vaccination involves administering a tumor antigen with the aim of stimulating tumor-specific immunity. Antigens could be delivered in the form of DNA or peptides, as well as tumor cells or antigen-pulsed DCs. To be considered an ideal tumor vaccine candidate, expression of the antigen must be restricted to the tumor or only minimally expressed elsewhere in the body.  Table 1 summaries a list of major candidate pancreas tumor-associated antigens for immune targeting. Additional synergistic help is added to elicit a more vigorous and effective immune response, such as cytokines and immunostimulating compounds. Vaccination against tumor antigens is an attractive approach to adjuvant treatment aftersurgery, when tumor-induced immune suppression is minimal [73–75].

### 3.1. Vaccines Using Whole Cells

The simplest vaccine approach that has been applied to cancer is the inoculation of patients with irradiated tumor cells. This approach remains a potent vehicle for generating antitumor immunity because tumor cells express all relevant candidate TAAs, including both known and unidentified. In the clinical setting, the use of autologous tumor cell depends on the availability of an adequate number of them. As only 10–15% of PC patients diagnosed are eligible for surgical, autologous pancreatic cancer cells may not be provided in most of the patients. Moreover, even if the patients are treated by surgical resection, it is difficult to prepare sufficient numbers of tumor cells due to the length of culture time and risk of contamination [76, 77]. To elude this problem, allogeneic tumor cell lines may be used instead of autologous tumor cells [78]. This strategy has many advantages: (1) specific TAAs do not need to be identified for vaccination, (2) allogeneic tumor cell lines are well characterized as TAAs source, (3) allogeneic tumor cell lines can grow well *in vitro*; thus, there is no limiting factor for preparation of tumor cells, (4) it is not necessary to determine HLA typing of patients and allogeneic tumor cells, because autologous DCs can process and present multiple TAAs from allogeneic tumor cells owing to cross-presentation in the context of appropriate MHC class I and II alleles [75, 79], (5) polyclonal antigen-specific T cells (CD4^+^/CD8^+^) can be generated, which may protect against tumor escape variants, and (6) the tumor cell vaccine platform can be easily modified. For example, tumor cells can be transduced to express immunomodulatory cytokines such as granulocyte macrophage colony-stimulating factor (GM-CSF), which has shown significant antitumor effect *in vivo *[80]. GM-CSF is an important growth factor for granulocytes and monocytes and has a crucial role in the growth and differentiation of DCs. In a phase I clinical trial, Jaffee et al. [80] used allogeneic GM-CSF-secreting whole-cell tumor vaccine for pancreatic cancer, based on the concept that the GM-CSF localization in the implanted tumor environment together with the shared tumor antigen expressed by the primary cancer would effectively induce an antitumor immune response. In this study two PC cell lines (PANC 10.05 and PANC 6.03) were used as the vaccine, both genetically modified to express GM-CSF and then irradiated to prevent further cell division. 14 PC patients who had undergone pancreatic duodenectomy eight weeks before were given variable doses of the vaccine intradermally. Three of the eight patients who received ≥10 × 10^7^ vaccine cells developed postvaccination delayed-type hypersensitivity (DTH) responses associated with increased disease-free survival time and remained disease free for longer than 25 months after diagnosis. Side effects were mainly limited to local skin reactions at the site of vaccination. 

In a recently completed phase II study 60 patients with resected pancreatic adenocarcinoma received five treatments of 2.5 × 10^8^ vaccine cells, together with 5-FU and radiotherapy [81]. The reported MS was 26 months, with a one- and two-year survival of 88% and 76%, respectively. In these two studies, a PC cell vaccine induced a CD8^+^ T cell response, specific to mesothelin, regardless of HLA match between the tumor vaccine and recipient—demonstrating that cross-priming had occurred [80, 82]. Mesothelin is a particularly promising cancer vaccine target owing to its low level of expression in nontumor tissues and high levels of expression in pancreatic as well as other cancers (i.e., ovarian) [83]. Laheru et al. [84] administrated GMCSF-secreting allogeneic PC cells in sequence with cyclophosphamide in patients with advanced pancreatic cancer. The approach showed minimal treatment-related toxicity and mesothelin-specific T cell responses. Moreover, combination of the vaccine and cyclophosphamide resulted in MS in a gemcitabine-resistant population similar to chemotherapy alone. It was also reported that combination of the vaccines and chemoradiation demonstrated an overall survival that compares favorably with published data for resected pancreas cancer [85].

Tumor cell vaccines have also been modified to express epitopes, which increase antibody-mediated uptake by DCs. Normally, MUC-1 expressed on tumors is immunogenic owing to overexpression and tumor-restricted hypoglycosylation [86]. The NewLink Genetics Corporation (IA, USA) has developed a whole-cell vaccine expressing MUC-1 modified to express *α*-gal epitopes, which is the focus of multiple clinical trials [87–90]. This vaccine takes advantage of anti-*α*-gal antibodies that are found in most people due to exposure to gastrointestinal flora, resulting in increased uptake of the vaccine in an antibody-dependent manner [91]. In murine models, the addition of such *α*-gal epitopes to a Muc-1^+^ PC whole-cell vaccine resulted in increased production of anti-Muc-1 antibodies, enhanced tumor-specific T cell responses, and increased survival after challenge with Muc-1^+^ B16 cells in *α*-gal knockout mice, previously sensitized to *α*-gal [92].

### 3.2. Peptide Vaccines

Peptide-based cancer vaccines are preparations made from antigenic protein fragments (called epitopes), that represent the minimal immunogenic region of antigens [93, 94], designed to enhance the T cell response, especially the CD8^+^. Induction of CTLs needs peptides derived from TAAs to be presented on the surface of APCs (antigens presenting cells), such as DCs, in the context of HLA molecules. The major advantages of peptide vaccines are that they are simple, stable, safe, economical, and do not require manipulation of patient tissues, whose availability may be limited. However, there are also several obstacles that limit the widespread usefulness of peptide vaccines: (1) a limited number of known synthesized short peptides cannot be available in many HLA molecules [95–97], (2) impaired function of APCs in patients with advanced pancreatic cancer [76, 98], (3) CTLs may be ineffective in reacting with PC cells downregulated by certain tumor antigens and MHC class I molecules, which may appear during the course of tumor progression [99], (4) regulatory T cells (Tregs) or MDSCs (myeloid-derived suppressor cells) in tumor environment produce immunosuppressive cytokines such as IL-10 and TGF-*β* [100]. 

Anyway, a number of peptide vaccines have undergone phase I/II clinical trials [12, 101], showing encouraging results, due to their ability to produce cancer-specific responses in PC patients (Table 2). In a phase I study, vaccination with a 100 mer peptide of the MUC-1 extracellular tandem repeat generated a MUC-1-specific T cell response in some PC patients with two of the 15 patients alive at 61 months [102]. Moreover, in a separate phase I clinical trial using the same peptide vaccine, the production of anti-MUC-1 circulating antibodies was detected in patients with inoperable PC, although no significant impact on survival was discovered [103].

In a phase I trial, Miyazawa et al. administered a peptide vaccine for human VEGF receptor, (VEGFR)2-169 epitope, in patients with advanced PC, in combination with gemcitabine, observing an antigen-specific DTH and VEGFR2-specific CD8^+^ cells in 61% patients, with an overall MS time of 8.7 months [101]. A randomized, placebo-controlled, multicenter, phase II/III study of this VEGFR2–169 peptide vaccine therapy, combined with gemcitabine, is currently underway in patients with unresectable advanced or recurrent PC [104]. In similar studies, a telomerase-based vaccine, consisting of the human telomerase reverse transcriptase (GV1001) peptide, was found to induce a telomerase-specific immune response in 63% of evaluable patients, as measured by DTH in unresectable PC. Those with a positive DTH were found to live longer than those that did not have a positive DTH [105]. In addition, augmented immune responses and prolonged survival were observed following vaccination of advanced PC patients with telomerase peptide and GM-CSF [105]. More recently, a phase III clinical trial was performed in which the effect of gemcitabine treatment on survival was compared with gemcitabine treatment in combination with GV1001 therapy in unresectable and metastatic PC patients [106]. However, the trial was terminated when no survival benefit was found. 

The most interesting results have come from studies of K-Ras-targeted peptide vaccines. Gjertsen et al. [77] first reported mutant K-ras peptide vaccines for PC. In a phase I/II trial involving 48 PC patients, they studied ras peptide in combination with GM-CSF, since native epitopes have relatively low immunogenicity [77]. Peptide-specific immunity was induced in 58% of patients. Of patients with advanced disease, those who responded to treatment showed increased survival compared to nonresponders. Recently, another group reported that vaccination of 24 PC patients with K-ras peptide in combination with GM-CSF proved to be safe without tumor regression [107]. In another pilot vaccine study, pancreatic and colorectal patients were vaccinated with K-Ras peptides containing patient-specific mutations. Three of the five PC patients displayed an antigen-specific immune response to a K-Ras [108]. Disease progression was observed in the two PC patients that did not respond to the vaccine, with the responders having no evidence of disease. Of the PC patients, a mean disease-free survival of 35.2 months and a mean overall survival of 44.4 months were observed. Such results with peptide vaccines are highly encouraging. 

The more attractive peptide-based vaccines may be synthetic long peptides to induce antigen-specific polyclonal CD8^+^ and CD4^+^ T cells [109]. Long synthetic peptides cannot bind directly on MHC class I or II molecules, but they need to be processed and presented by DCs. So, the long peptide vaccines can present MHC class I- and II-restricted epitopes for long time, thus eliciting both CD4^+^- and CD8^+^-mediated immune recognition [110] and probably inducing a robust therapeutic T cell response. In a phase I study using long synthetic mutant ras peptides, Wedén et al. [111] treated 23 patients who were vaccinated after surgical PC resection. Long-term immunological memory responses to the vaccines were observed. Strikingly, 10-year survival was 20% (four patients out of 20 evaluable) versus zero (0/87) in a cohort of nonvaccinated patient treated in the same period.

To increase the immunogenicity of peptide vaccines, some groups have mutated key anchor residues in the peptides such that binding to MHC-I molecules, and consequently the presentation to CD8^+^ T cells, is increased. This is particularly important when vaccinating against TAAs, as they are often weak or only intermediate binders to HLA molecules [112–116]. An MUC-1 peptide vaccine modified in this way was shown to enhance production of IFN-*γ* from patient and normal donor T cells. MUC-1-specific T cell clones, generated by stimulation with this peptide, could lyse targets pulsed with native Muc-1 epitope as well as HLA-A2^+^ MUC-1^+^ human tumor cells *in vitro* [117]. Notably, one case has been reported in which vaccination with a modified HLA-A2-restricted survivin peptide resulted in remission of liver metastasis in one PC patient [17].

Another approach in cancer peptide-vaccination consists in using personalized peptide vaccines based on the tumor-antigen epitopes that are most immunogenic for a particular patient. In a phase I clinical trial, Yanagimoto et al. applied this strategy, in combination with gemcitabine therapy, to pancreatic cancer. Prior to vaccination, T cells from patient PBMCs were screened against a panel of tumor antigen-derived peptides. Patients were vaccinated only with the peptides to which they had a response [12]. An increase in tumor antigen-specific T cell responses was observed from the 13 evaluable patients with no correlation to clinical responses or humoral responses following vaccination, although 11 patients experienced either reduction in tumor size. Median survival time was 7.6 months. A similar phase II study was published in 2010 by the same group, showing an MS time of 9 months and a 1-year survival of 38% [118].

### 3.3. DNA Vaccination

Vaccination with DNA represents a simple vehicle for *in vivo* transfection and antigen production. A DNA vaccine is composed of a plasmid DNA that encodes for a TAA under the control of a mammalian promoter and can be easily produced in the bacteria [119]. It can be administered to humans via intramuscular injection with or without electroporation. Compared with cell-based vaccines, this vaccination strategy offers more advantages; in fact, while cell-based vaccines become less effective over time because the induced immune system recognizes them as foreign and quickly destroys them, DNA vaccines can provide prolonged antigen expression, leading to amplification of immune responses and inducing memory responses against weakly immunogenic TAAs. Moreover, as DNA might be taken up by cells and the encoded antigen is processed through both endogenous and exogenous pathways, DNA vaccines administered via intramuscular injection allow for an immune response to multiple potential epitopes within an antigen to be generated regardless of the recipient's MHC profile [120]. Actually DNA vaccines are ongoing trials in different tumors [121–123] and being studied in murine models of pancreatic cancer. In a murine PC study, an MUC-1 DNA vaccine was able to induce a significant MUC-1-specific CTL response and had both prophylactic and therapeutic effects in tumor-bearing mice [124]. Similarly, in another PC murine model, vaccination with either murine or human full-length survivin DNA generated an antitumor-specific response, increased infiltration of tumor with lymphocytes and increased survival [125]. Furthermore, Gaffney et al. studied the mesothelin DNA vaccine in combination with the antiglucocorticoid-induced TNF receptor antibody (anti-GITR) in mice with syngeneic mesothelin-expressing pancreatic cancer [126]. 50% of animals treated with mesothelin were tumor free 25 days after tumor injection compared to 0% of nontreated mice. This increased to 94% with the addition of anti-GITR. The agonist anti-GITR served to enhance T cell-mediated response of the vaccine [127, 128].

### 3.4. Antigen-Pulsed DCs

Antigen-specific T cell responses are initiated by DCs. They capture antigens secreted or shed by tumor cells and present peptides in association with the MHC class I and II molecules. This results in the expression and upregulation of cytokines and costimulatory molecules which in turn stimulate CD4^+^ and CD8^+^ T cells to mount an antitumor response [129]. Therefore, a major area of investigation in cancer immunotherapy involves the design of DCs-based cancer vaccines [130]. Autologous DCs can be used in tumor vaccination (1) pulsed with synthetic peptide derived from the known tumor antigens [131], tumor cell lysates [132], or apoptotic tumor cells [133], (2) transfected with whole-tumor mRNA [134] or with mRNA or cDNA of a specific antigen [135] and (3) fused with tumor cells to induce antigen-specific polyclonal CTL responses [136]. 

DC-based vaccines have been used in different PC studies. Schmidt et al. intratumorally vaccinated with whole tumor mRNA transfected DCs and found an antitumor-specific immune response and significantly decreased tumor volume in a murine PC model [137]. Apoptotic PC lysates have also been evaluated as a source of antigens and have been demonstrated to elicit stronger antitumor lytic activity when used to stimulate autologous human CD8^+^ T cells *in vitro* compared with those stimulated with tumor lysate-pulsed DCs [138]. In cases in which an immunogenic tumor antigen is known, autologous DCs have been transfected with or virally transduced to express, the mRNA or cDNA of a specific tumor antigen (Table 3). A vaccine consisting of liposomal MUC1-transfected autologous DCs was evaluated in a clinical phase I/II trial. In MUC1 peptide-loaded DC vaccines in PC patients following resection of their primary tumors, four of the 12 patients followed for over four years were alive, all without evidence of recurrence [139]. Moreover, MUC1-specific immune responses were also observed even in patients with pretreated and advanced disease, following immunization with DCs transfected with MUC1 cDNA [140]. This technique does not require that the exact immunogenic epitopes of the antigen be identified, as full-length protein is transfected. 

In another study, three patients with resected PC following neoadjuvant chemoradiotherapy were given monthly injections of autologous, monocyte-derived DCs loaded with the mRNA of CEA for six months [141]. No toxicities were reported, and all patients remained disease free for more than 30 months from diagnosis.

Pulse can also be performed with peptides from multiple tumor antigens, as was performed in a Phase I clinical study by Carbone et al. Patients with various cancers, including pancreatic cancer, immunized with p53 and K-ras peptide-pulsed PBMCs, saw increased survival [142]. In addition, autologous DCs, virally transduced to express IL-12, have also been used in cancer treatment. One PC patient receiving this treatment had a partial response in studies by Mazzolini et al. [143]. As the treatment DCs were not loaded with tumor antigens, cross-presentation of tumor antigens must have occurred. Moreover, DCs have been fused with tumor cells to induce antigen-specific polyclonal CTL responses [136]. In the DC/tumor cell fusion approach, whole TAAs including those known and those yet unidentified are processed endogenously and presented by MHC class I and II pathways in the context of costimulatory signals [144–146]. In particular, this technique has been used to treat mice in a PC model, resulting in the generation of CD8^+^ T cells with tumor-specific cytolytic activity and tumor rejection [147].

## 4. Conclusion

Pancreatic cancer is a dismal disease that has a high morbidity and mortality, and at present there are not effective chemotherapeutic treatments, especially for patients with advanced and metastatic diseases. For all these reasons Alphais of prime importance to investigate new pancreatic cancer treatments. In this paper we have analyzed the various strategies of the immunotherapeutic approach, some of which are still used in animal models; others are already being exploited in clinical trials. Immunotherapy is certainly a promising treatment for pancreatic cancer, because it is highly specific for cancer cells and therefore without the side effects associated with traditional chemotherapy. But at the moment there are not antigens expressed only by PC cells; in fact the antigens used as the target of immunotherapic treatments are self-protein or overexpressed [65, 148–150] in tumor cells or present in acetylated form [151], with the risk of autoimmune phenomena. However, the data obtained in different clinical trials showed an increase in the survival of patients treated with PC immunotherapy alone [72, 105, 108, 111] or in combination with chemotherapy treatments [12, 54, 101], with very minimal autoimmune manifestations. In conclusion we can say that immunotherapy may be included among the future treatments for pancreatic cancer, especially for inoperable patients, but for the effectiveness of this innovative treatment is essential to overcome some obstacles: (a) finding specific markers for pancreatic cancer cells, (b) mitigating the immune suppressive effects of tumor cells, (c) early diagnosis of the tumor so as to act in a timely manner before the cancer spreads in other locations.

## Figures and Tables

**Table 1:** Pancreatic cancer-associated antigens for immune targeting.

PC-associated antigens	Characteristics and functions	Location	Tumor expression	References
CEA	Glycoprotein, normally expressed only in oncofetal tissues. Functions as cell-adhesion molecule. First tumor antigen to be described.	Cell surface (GPI-linked)	Overexpressed	[148, 152]
Her2-neu	A receptor tyrosine kinase, member of the EGF-receptor family, involved in cell growth and differentiation.	Transmembrane	Over-expressed	[149]
MUC-1	Type I transmembrane glycoprotein, expressed on apical surface of ductal and glandular epithelial cells at low levels. Extracellular domain has a polypeptide core with multiple tandem repeats of 20 aminoacids.	Transmembrane	Over-expressed, hypo-glycosylation	[37, 153]
P53	Tumor suppressor that regulates cell cycle. Normally inhibits survival at the transcription level and can initiate apoptosis if DNA damage is irreparable.	Intracellular	Mutated self	[154, 155]
Survivin	Member of IAP family. Inhibits caspase activation, is found in most human tumors and fetal tissue, but is completely absent in terminally differentiated cells.	Intracellular	Over-expressed	[150]
K-ras	Mutated form of ras, a GTPase important for cell proliferation, differentiation, and survival.	Intracellular	Mutated self	[156]
Telomerase	Ribonucleoprotein that is responsible for RNA-dependent synthesis of telomeric DNA. TERT is its catalytic subunit.	Intracellular	Over-expressed	[65]
VEGFR2	A tyrosine kinase and member of platelet-derived growth factor family. Receptor for VEGF with functions in blood vessel development.	Transmembrane	Over-expressed	[157]
Mesothelin	GPI-linked glycoprotein, expressed on the surface of mesothelial cells lining the pleura, peritoneum, and pericardium at low levels. Binding partner of CA125/MUC16.	Cell surface (GPI-linked)	Over-expressed	[27, 158]
Alfa-enolase	Glycolytic enzyme that also acts as a surface plasminogen receptor. Is found in a variety of tissue, on the cell surface as well as within the nucleus and cytosol.	Cell surface, Intracellular	Over-expressed, post-translational modified (i.e., acetylated)	[21, 151, 159]

**Table 2:** Peptide vaccines-based clinical trial.

Peptide (Adjuvant)	Combination	Patients enrolled	Phase of the study	Clinical results	References
100 mer MUC1 (SB-AS2 adjuvant)		16 with resected or locally advanced PC	I	Detectable MUC1-specific humoral and T-cell responses were detected in some patients.	[102]
100 mer MUC1 (incomplete Freund's adjuvant)		6 with advanced PC	I	One patient showed a tendency for increased circulating anti-MUC1 IgG antibody.	[103]
VEGFR2-169	Gemcitabine	21 with unresectable PC	I	Specific cytotoxic T lymphocytes (CTL) reacting to the VEGFR2-169 peptide were induced in 11 (61%) of the 18 evaluable patients. The disease control rate was 67%, and the median overall survival time was 8.7 months.	[101]
Telomerase (GM-CSF)		48 with advanced PC	I/II	Immune responses were observed in 24 of 38 evaluable patients. One-year survival for the evaluable patients in the intermediate dose group was 25%.	[105]
Mutant K-ras (GM-CSF)		10 with resected and 38 with advanced PC	I/II	Immune response to the peptide vaccine showed prolonged survival compared to nonresponders. K-ras-specific T cells were selectively accumulated in the tumor.	[77]
Mutant K-ras (GM-CSF)		24 with resected PC	Pilot study	Vaccination proved to be safe and tolerable with however no elicitable immunogenicity and unproven efficacy.	[107]
13 mer mutant ras		5 with PC and 7 with colorectal cancer	II	This vaccine is safe, can induce specific immune responses, and it appears to have a positive outcome in overall survival. The five pancreatic cancer patients have shown a mean disease-free survival (DFS) of 35.2+ months and a mean overall survival (OS) of 44.4+ months.	[108]
Mutant ras long peptide		23 with resected PC	I	17 of 20 evaluable patients (85%) responded immunologically to the vaccine. Ten-year survival was 20% (four patients out of 20 evaluable).	[111]
Surviving		1 with liver metastasis of PC refractory to gemcitabine	Case report	The patient initially underwent partial remission of liver metastasis which proceeded after 6 months into a complete remission with duration of 8 months.	[17]
Personalized peptide vaccine	Gemcitabine	11 with advanced PC	I	The 6- and 12-month survival rates for patients who received >3 vaccinations (*n* = 10) were 80% and 20%, respectively.	[12]

**Table 3:** DC-based vaccines clinical trial.

DC-based vaccines	Patients enrolled	Phase of the study	Clinical results	References
MUC1 peptide-loaded DC	12 with resected pancreatic and biliary cancer	I/II	4 of the 12 patients followed for over four years were alive	[139]

DC transfected with MUC1 cDNA	10 with advanced breast, pancreatic, or papillary cancer	I/II	4 patients showed a 2- to 10-fold increase in the frequency of MUC1-specific IFN-gamma-secreting CD8^+^ T cells.	[140]

mRNA CEA-loaded DC	3 with resected PC	Pilot study	The immunizations were well tolerated without evidence of adverse events. All patients were alive without evidence of disease at more than 30 months from the original diagnosis.	[141]

Peptides (mutant p53- and k-ras-loaded DC	39 patients with several types of cancer (lung, breast, pancreatic, ovarian, colon, others)	I	10 (26%) of 38 patients had detectable CTL against mutant *p53* or *K*-*ras*, and 2 patients were positive for CTL at baseline. Positive IFN-*γ* responses occurred in 16 patients (42%) after vaccination, whereas 4 patients had positive IFN-*γ* reaction before vaccination. Cellular immunity to mutant *p53* and *K*-*ras* oncopeptides is associated with longer survival.	[142]

DC engineered (secreting IL-2)	17 patients with several types of cancer (3 metastatic pancreatic, 5 colorectal, 9 liver, cancer)	Pilot study	Treatment was well tolerated. DC treatment induced a marked increase of infiltrating CD8^+^ T lymphocytes in three of 11 tumor biopsies analyzed. A partial response was observed in one patient with pancreatic carcinoma.	[143]
